# Long-Term Evaluation of Visual Outcomes and Patient Satisfaction after Binocular Implantation of a Bioanalogic Lens

**DOI:** 10.1155/2021/5572384

**Published:** 2021-05-07

**Authors:** Sylwia Wagner, Grzegorz Wagner, Ewa Mrukwa-Kominek

**Affiliations:** ^1^Department of Ophthalmology, Professor Kornel Gibinski University Hospital Centre, Medical University of Silesia, 35 Ceglana St, Katowice 40514, Poland; ^2^Department of Biochemistry, Faculty of Medical Sciences in Zabrze, Medical University of Silesia, Katowice 40055, Poland; ^3^Departament of Ophthalmology, Faculty of Medical Sciences in Katowice, Medical University of Silesia, Katowice 40055, Poland

## Abstract

**Purpose:**

Long-term evaluation of the visual refractive outcomes and the quality of life after implantation of the WIOL-CF (Medicem, Czech Republic) in both eyes.

**Design:**

retrospective, nonrandomized noncomparative case series.

**Methods:**

50 eyes of 25 patients, including 11 women (44%) and 14 men (56%). The age range of the patients was 38 to 77 years (mean age 55.48 ± 10.97 years). All patients underwent bilateral implantation of the WIOL-CF. Exclusion criteria were previous ocular surgeries except for cataract surgery and refractive lens exchange, irregular corneal astigmatism of >1.0 diopter, and ocular pathologies or corneal abnormalities. Postoperative examinations were performed at 14 days and 3, 6, 12 months of surgery; the last follow-up was between 24 and 36 months after the procedure. All exams included manifest refraction, monocular uncorrected visual acuity (UCVA) and distance-corrected visual acuity (DCVA) in 5 m (Snellen), monocular uncorrected visual acuity in 70 cm and 40 cm (Jeager) and binocular UCVA, DCVA in 5 m, 70 cm, and 40 cm, binocular contrast sensitivity (CS) under photopic conditions, binocular defocus curves, high-order aberrations, quality-of-vision VF-14 questionnaire, and spectacle independence.

**Results:**

Significant improvement in monocular visual acuity at all distances was demonstrated; the mean postoperative spherical equivalent was 0.32 ± 0.45D. The postoperative means of binocular distance UCVA and BCVA were also improved (*p* < .001) and so were the mean uncorrected intermediate VA (2.053 ± 1.268) and near uncorrected VA (2.737 ± 1.447). There was a significant improvement in contrast sensitivity at all spatial frequencies and higher-order aberration, compared to preoperative results.

**Conclusions:**

The evaluation of a WIOL-CF showed good *distance*, *intermediate*, and *near* visual acuity. Contrast sensitivity increased after surgery in all spatial frequencies. Patient satisfaction was high despite some optical phenomena. The rate of postoperative spectacle independence also turned out high. *Financial Disclosure*. No author has a financial or proprietary interest in any material or method mentioned.

## 1. Introduction

Cataract surgery is meant to obtain the best visual acuity in the eyes operated on. Patient selection is crucial in achieving success with premium IOLs. Before the procedure, it is necessary to talk to the patient in order to determine their expectations and lifestyle needs [1,2]. If the patient opts for premium intraocular lenses, their selection applies to distance, intermediate, and near vision. We do not have knowledge or lens that would fully replace the natural one. Hence, “the main goals of intraocular lens technology are to achieve proper balance between good vision at all distances and spectacle independence as well as lower contrast sensitivity and tolerance of negative light phenomena” [3]. Patients should be informed about possible adverse effects and especially about potential optical aberrations. Although some of these symptoms may partially resolve over time through neuroadaptation, patients must be aware they might persist [4]. The age-related decrease in accommodative amplitude becomes symptomatic around the age of 45 and is referred to as presbyopia. Factors that may contribute to presbyopia are lens hardening, ciliary muscle and choroid aging, loss of elasticity of the lens capsule, or lens growth throughout life [5]. Pseudoaccommodation has been associated with several ocular characteristics such as pupil size, anterior chamber depth (ACD), age, postoperative astigmatism, the effect of axial length (AL) on IOL shift, higher corneal aberrations, higher ocular aberrations, and corneal multifocality [6]. Despite the loss of accommodation caused by ciliary muscle weakening, pharmacological stimulation by instillation of pilocarpine as well as *in vivo* and *in vitro* studies using ultrasound biomicroscopy and Magnetic Resonance Imaging (MRI) showed that the function of the ciliary body persisted over the years, even in pseudophakic patients [7]. The effort to focus the near vision in pseudophakic eyes causes convergence and contraction of the pupils and hence ciliary body activation [8]. Pseudoaccommodation with monofocal IOLs ranges between 0.7 and 5.1 D, depending on the method of assessment, with a mean of about 2D [9]. Presbyopia can be corrected with contact lens or spectacles; however, its surgical correction remains a significant challenge for refractive surgeons. Accommodative and pseudoaccommodative surgical strategies for presbyopia include extraocular (corneal or scleral) or intraocular (removal and replacement of the crystalline lens or some type of crystalline lens treatment) interventions. Each has its own benefits and limitations and may involve some degree of compromise between the distance and near visual acuities [10]. Presbyopia-correcting IOLs can be divided into 3 broad categories: MF IOLs (including diffractive or refractive designs), extended depth-of-focus (EDOF) IOLs, and accommodative IOLs (intracapsular or sulcus placed) [4]. Hence “in contrast to multifocal (MF) IOLs, EDOF lenses create a single elongated focal point, rather than several foci, to enhance depth of focus. In this way, EDOF IOLs aim to reduce photic phenomena, glare, and halos, which have been reported in MF IOLs. A potential disadvantage is a decrease of retinal image quality if the amount of the aberrations is excessively increased” [11]. According to Kohnen and Suryakumar review despite differences in IOL design among models, EDOF IOLs provide good to excellent visual acuity at distance, improved intermediate visual acuity compared with monofocal IOLs, and functional near visual acuity [12].

The Wichterle IOL-continuous focus (WIOL-CF) (Medicem, Kamenné Žehrovice, Czech Republic) is a bioanalogic lens with one-piece polyfocal optics. Accommodation is just one of the three mechanisms ensuring vision at all distances. The other two mechanisms are represented by polyfocality (providing high depth of focus enabled by hyperbolic optics) and pseudoaccommodation enabled by a combination of polyfocality and pupillary reflex [13]. Additionally, large 8.9 mm and hyperbolic optics (without zones) improves lens centricity [14]. All of the patients should underwent a complete preoperative evaluation [15]. A correct intraocular lens must be selected for the patient, who should also be aware that artificial lenses do not work like their natural equivalents.

## 2. Patients and Methods

The study comprised 50 eyes of 25 patients including 11 women (44%) and 14 men (56%). The mean age was 55.48 ± 10.97 (SD) years (range 38–77 years). The study protocol was approved by the local ethics committee and was in accordance with the Declaration of Helsinki. All patients gave their written informed consent and underwent bilateral implantation of the WIOL-CF. The value of the WIOL-CF lens was calculated based on the preoperative examinations (according to the WIOL-CF calculator); the mean power of the lens was 23.07 (±2.17) D. The study included patients who had undergone phacoemulsification and implantation of WIOL-CF. Exclusion criteria were previous ocular surgeries including cataract surgery and refractive lens exchange, laser refractive surgery, radial keratotomy, irregular corneal (keratometric) astigmatism higher than 1.0 diopter (D), ocular pathologies, corneal abnormalities, and endothelial cell count below 2000/mm2. The qualifying examination included uncorrected and corrected distance acuity (Snellen charts at a distance of 5 m), uncorrected near and intermediate vision (Jeager charts 40 cm, 70 cm), corneal topography (TMS, Tomey, Germany), intraocular pressure (tonometry), optical biometry-optical ultrasonography with the IOL Master apparatus (Zeiss, Germany), measurement of the anterior chamber depth (OCT-Casia, Tomey, Germany), corneal endothelial cells density (CS, Italy), contrast sensitivity test (FVA, USA), higher-order aberrations (WASCA, Zeiss, Germany), assessment of the anterior and posterior eye segments, and the patient's satisfaction questionnaire (VF-14). Follow-up examinations were performed on day 14 and 3, 6, and 12 months after the procedure; the last follow-up was between 24 and 36 months of surgery. It included an evaluation of the uncorrected and best-corrected distance (mono- and binocular), near, and intermediate visual acuity and contrast sensitivity measurements. Binocular defocus curve and patient satisfaction questionnaire were also obtained. The contrast sensitivity was assessed monocularly under photopic conditions (luminance level 85 cd/m2, no glare) with undilated pupils. The quality of vision after surgery was assessed using the FACT test (Stereo Optical Co., Inc., USA) including the VF-14 test (14-item Visual Function Index) and spectacle independence questionnaire. Safety and efficacy indices were calculated postoperatively. The defocus curve was examined during the postoperative observation period using long distance (5 m) Snellen charts. The examination was performed binocularly for defocus levels ranging from +3.0 to −4.0 diopters, in 0.5-diopter steps.

Intraocular lens: a bioanalogic, polyfocal, hydrogel lens, WIOL-CF (Witerchle Intraocular Lens- Continous Focus) by Medicem Technology, Czech Republic, was used. The lens is foldable, one-piece, devoid of haptics and positioning holes. It has one optical diameter of 8.6–8.9 mm, made of a synthetic hydrogel, WIGEL. The lens can be biconvex, flat-convex, or convex-concave, depending on the dioptric power of the lens. The refractive power of the lens decreases from the center to the circumference and so does the lens thickness, which varies from 1.7 mm in the center to 0.8 mm in the circumference. Optical power is from +15.0 to +30.0 D in 0.5 D steps. Nominal refractive index is 1.43, and water content is 42 ± 2%. The lens increases its volume from 7 mm³ to 9 mm³ after implantation. Patients were advised to limit the use of spectacles for approximately 3 months in order to take full advantage of the lens focusing ability. This facilitates the neuroadaptation process.

### 2.1. Surgical Technique

All surgeries were performed by one surgeon. Local anesthetic drops were instilled and standard phacoemulsification technique was used. A clear corneal incision of 2.8 mm was made on the steep axis of the cornea, and an anterior curvilinear capsulorhexis of 5.5 mm was performed with extreme care to ensure central lens position. The partly dehydrated hydrogel WIOL-CF lens was injected through a 2.8 mm incision. In its dehydrated state, the lens was smaller in size and much stronger than in its fully hydrated state and could be folded prior to the implantation. Following insertion, the lens unfolded inside the capsule and became gradually hydrated with eye fluids. Full hydration took 24 to 48 hours with the lens filling most of the capsular space. Osmotic pressure related to ongoing lens hydration secured the adhesion of the lens to the posterior capsule and prevented lens dislocation. After surgery, all patients received an antibiotic and corticosteroid, five and four times daily, respectively, with a 1-month taper regimen.

### 2.2. Statistical Analysis

The database was prepared in Excel 2007 (Microsoft Office USA). Statistical evaluations were performed using the STATISTICA PL version 13. The Shapiro-Wilk test was used to test for the normality of data distribution. When parametric analysis was possible (parameters before and after the treatment), then, after additional checking of the homogeneity of variance and sphericity, the means of parameters were compared using the ANOVA test. When parametric analysis was not possible, the Friedman test for multiple paired samples was used. An appropriate *t*-test was used to compare variables with normal distributions. When data were not normally distributed, the nonparametric Wilcoxon test was used for dependent variables and the nonparametric Mann-Whitney *U* test for independent variables. The level of significance was set at *p* < 0.05.

## 3. Results

**Figure 1:**
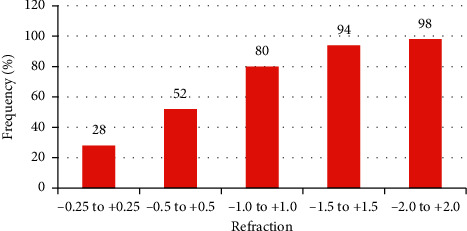
Distribution of the postoperative spherical equivalent (SE) after surgery in the analyzed group.

The mean age of the patients was 55.48 ± 10.97 (range 38–77 years). All patients underwent cataract surgery in both eyes. Spherical equivalent (SE) changed from −0.145 (±1.887) D preoperatively to 0.321 (±0.448) D at the last control. The mean cylindrical value decreased from −0.02 (±0.416) Dcyl before treatment to −0.013 (±0.292) Dcyl at the last control. It was found that only 6% of eyes exceeded the range of ±1.5 D from emmetropia (target postoperative refraction was 0.0 D), and 80% of SE eyes were within ±1.0 D (Table 1 and Figure 1).

**Figure 2:**
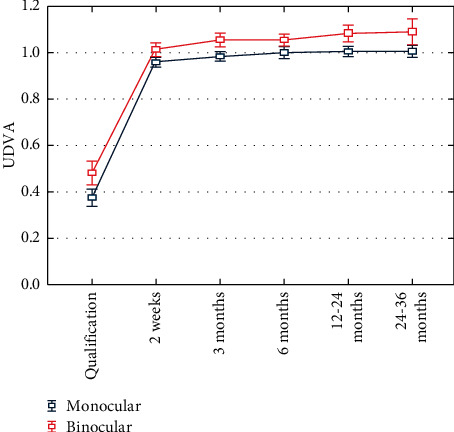
Comparison of mean changes in monocular and binocular UDVA before and after surgery, including SD.

Significant improvement in VA has been shown at all distances: mean UDVA was 1.008 (±0.168), BCDVA 1.056 (±0.171), and UIVA 2.538 (±1.62), at the last control (Table 2). Significant improvement in binocular VA has been shown at distances: mean UDVA was 1.089 ± 0.251, mean BCDVA was 1.132 ± 0.25, and mean BIVA was 1.316 ± 0.582 at the last control (Table 3). A comparative analysis of monocular and binocular acuity of the uncorrected distance was performed using the nonparametric Mann-Whitney *U* test, which showed better binocular visual acuity than monocular visual acuity, statistically significant for the observation period from 3 months to 24–36 months after the procedure, and the significance levels for these postoperative periods were consecutively: *p* = 0.02, *p* = 0.036, *p* = 0.028, *p* = 0.012 (Figure 2).

**Figure 3:**
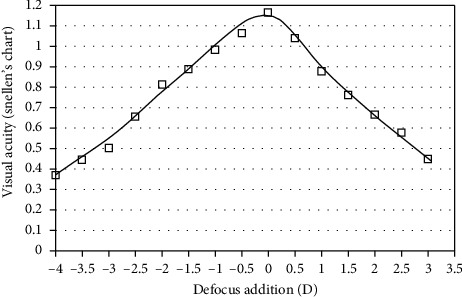
Defocus addition (D) visual acuity (Snellen).

At the three main VA levels of the defocus curve corresponding to the distance, near, and intermediate vision distances, the best visual acuity results (VA 1.165 and 0.887) were obtained for the 0.00D and −1.5D levels, simulating distances for 5 m and 70 cm. VA worsened (0.652) slightly for the −2.5 D (40 cm) level (Figure 3).

The coefficients of effectiveness and safety were greater than 1.0 and remained at a similar level in subsequent follow-up examinations in all of the eyes. Both the effectiveness of the procedure and the level of safety were assessed by calculating the following coefficients: effectiveness (ratio of the best uncorrected postoperative visual acuity to the best-corrected visual acuity before surgery) and safety (ratio of visual acuity in the best eyeglass correction after the procedure to visual acuity in the best correction before the procedure).

**Figure 4:**
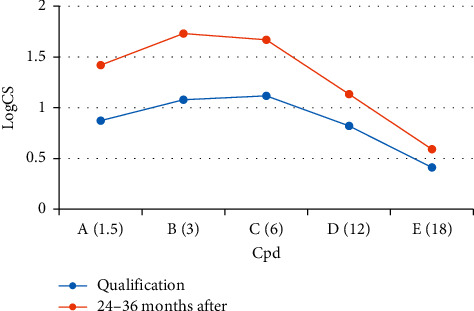
The mean contrast sensitivity function with different spatial frequencies.

There was a significant improvement in the CS before and after surgery, performed in photopic conditions in the range of all spatial frequencies; *p* value: A *p* = 0.018; B *p* = 0.015; C *p* = 0.015; D *p* = 0.019; E *p* = 0.0009 (Table 4 and Figure 4). There was a significant improvement in the higher-order aberration values (TA, HOA) compared to the pretreatment test (Table 5).

**Figure 5:**
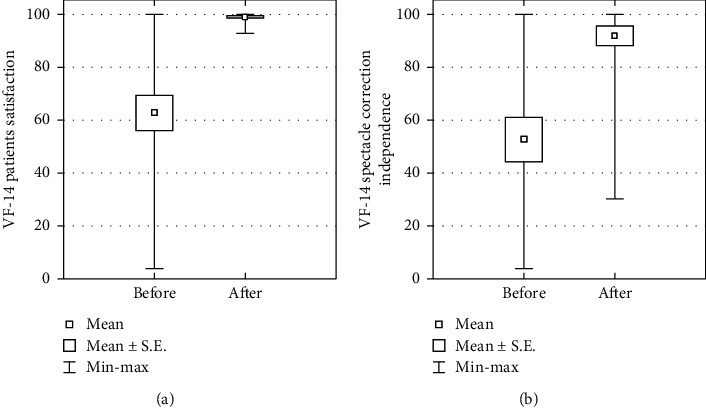
Vision satisfaction and independence from glasses before and after surgery.

No eye had intraoperative complications. Postoperative slit lamp examination in mydriasis showed well-centered IOLs in the capsular bag in all eyes. 12% of eyes developed PCO, and 2 (6% of eyes) patients underwent laser capsulotomy. 5 patients experienced photo-optical phenomena (halo, glare) which persisted until the last control. Despite this, the patients did not wish to remove the WIOL-CF lens due to good visual acuity. Subjective analysis of the visual function revealed a high degree of patient satisfaction with vision after surgery. The degree of difficulty in performing daily activities decreased, according to the patients, by an average of 36.28% after the procedure, and the necessity of using eyeglass correction to perform these activities decreased by an average of 39.03% (Figure 5).

## 4. Discussion

We present a long-term analysis of 25 patients, who underwent a binocular procedure of WIOL-CF lens implantation. The mean age was 55.48 (±10.97) years. The group size was the same as in the study by Pallikaris et al. while the mean age was lower [14]. Postoperatively, the magnitude of the cylindrical component decreased to −0.013 (±0.292) D; the ultimate spherical equivalent (SE) was 0.321 (±0.448) D. Only 6% of the eyes exceeded the range of ±1.5 D of emmetropia (target postoperative refraction was 0.0 D); 80% of eyes were within ±1.0 D. The only report on the postoperative spherical equivalent of refractive error after WIOL-CF lens implantation is the study by Pallikaris et al. [14], whose results were better compared to ours; the SE was −0.24 (±0.65) D, and 100% of the eyes were within ±1.0 D at one year after the procedure. Nevertheless, we also observed a significant improvement in monocular UDVA and BCDVA. Monocular UDVA was 0.6 and 0.9 (or better) during the postoperative and last follow-ups, respectively. This is the first comparative analysis of monocular and binocular UDVA; a significantly higher binocular visual acuity starting from the 3rd month of observation and until the last postoperative follow-up was shown compared to monocular vision. Kretz et al. found a significant VA improvement after implantation of trifocal AT LISA tri 839MP (Carl Zeiss, Germany). They concluded that binocular outcome was better than monocular results for all distances [16]. Pallikaris et al. reported the mean results in 25 patients (50 eyes) after binocular cataract surgery with the implantation of bioanalogic lenses: one-year UDVA and BCDVA were 0.074 (±0.19) and 0.082 (±0.13) logMAR, respectively. As in our study, both parameters were significantly different (*p* < 0.05) compared to presurgery visual acuity [14]. Similar results were also obtained by Studeny et al., who analyzed the impact of binocular WIOL-CF lens implantation in 48 patients (96 eyes). Six months after the procedure, the mean monocular UDVA was 0.074 (±0.108) logMAR, and BCDVA was 0.047 (±0,125) logMAR. The mean binocular UDVA was 0.022 (±0.053) logMAR and BCDVA was 0.008 (±0.024) logMAR [13]. Siatiri et al. also revealed a significant improvement (*p* = 0.002) in the mean BCDVA (from 0.2 (±0.14) to 0.01 (±0.09) logMAR) after cataract surgery combined with WIOL-CF lens implantation in 20 patients (40 eyes). However, the follow-up was shorter than in this study (13.10 (±5.52) months) [17]. Lower results were presented by Han et al. and Hyung et al. [18,19]. It should be noted though that study comparisons should be made cautiously due to differences in study design and patient populations. Nevertheless, previous studies evaluating bioanalogic IOLs demonstrated comparable *distance*, *intermediate*, and *near* visual acuities. VA outcomes after WIOL-CF were similar to values obtained after the implantation of multifocal, trifocal, and accommodative lenses. Marques and Ferreira compared visual outcomes after implantation of two types of trifocal diffractive lenses: Finvision Micro F (Physiol, Belgium) and AT LISA tri 839MP (Carl Zeiss, Germany). After 3 months of observation, the UDVA deviation was at least 0.3 logMAR in 30 eyes (100%) in patients with Finvision Micro F IOL and in 29 eyes (97%) in the AT LISA tri 839MP IOL group [20]. Jonker et al. reported the mean monocular UDVA of 0.09 (±0.16) logMAR, and the binocular UDVA of −0.01 (±0.11) logMAR in the group of 29 eyes with a binocular Finvision Micro F trifocal lens after 6 months of age [21]. According to Kohnen et al., the implantation of binocular trifocal lenses AT LISA tri839MP (Carl Zeiss) yielded higher UDVA than in the cited studies; the value obtained in a group of 27 patients at three months of surgery was −0.06 (±0.1) logMAR at 3 months after surgery [22]. Alio et al. assessed the characteristics of the Lumina accommodating lens (AkkoLens, The Netherlands). The UDVA was 0.04 (±0.11) logMAR, and 100% of eyes achieved a BCDVA of 0.1 logMAR after 12 months [23]. In a comparative study of 4 types of IOLs, Pedrotti et al. evaluated VA in 55 patients with TecnisSymfony ZXR00 lens (Abbott Medical Optics, USA). UDVA was −0.04 (±0.09) logMAR at 6 months after surgery, i.e., statistically better than in the other lens groups (monofocal Tecnis ZCB00, Abbott Medical Optics and multifocal Restor +2.5 D and Restor +3.0 D, Alcon) [24].

Our study demonstrated an improvement in uncorrected near vision acuity. The pre- and postoperative mean UNVAs were 5.158 (±4.371) and 3.359 (±1.769), respectively. 58.95% of eyes achieved UNVA of J3 or better. Higher UNVA scores including 85% of J3 were reported by Siatiri et al. [17]. Pallikaris et al.'s results were comparable to those of Siatiri; the uncorrected intermediate and near visual acuities were J2 (Snellen 20/25) or better in 72% of the eyes [14]. The results of our study are comparable to those of Studeny et al., where the mean postsurgery UNVA was 0.328 (±0.146) logMAR, and the BCNVA was 0.139 (±0.107) logMAR [13]. Marques and Ferreira presented the outcomes of 2 types of trifocal lenses; UNVA was at least 0.3 logMAR in all patients in both groups [20]. In the study by Alio et al., the mean monocular UNVA was 0.26 (±0.15) logMAR (at 40 cm) in the group of patients with Fine Vision IOL [25]. Similar results were obtained with trifocal lenses (Finevision Micro F) by Jonker et al.; the monocular UNVA was 0.25 (±0.17) logMAR (at 40 cm), and the binocular UNVA was 0.15 (±0.13) logMAR [21]. In the study by Kohnen et al., binocular trifocal lens implantation led to mean binocular UNVA of 0.04 (±0.1) logMAR [22]. UNVA of 0.07 (±0.08) logMAR and BCNVA of 0.1 logMAR were obtained in 90.32% of eyes at 12 months after cataract surgery and binocular Lumina accommodative lens implantation [23].

Regarding intermediate vision, a significant postoperative improvement in monocular UIVA was obtained; 60.53% of the eyes achieved UIVA of J2 or better. Other authors obtained insignificantly higher results in patients with bioanalogic lens. UIVA was J2 or better in 72% of the patients according to Pallikaris et al., while Studeny et al. [13, 14] reported mean UIVA of 0.178 (±0.123) logMAR. The 24-month binocular UIVA in our study was 2.053 (±1.268). The abovementioned results are lower than those obtained by Studeny et al. (the binocular UIVA was 0.10 (±0.105) logMAR after the procedure) [13]. However, comparable results were obtained by Jonker et al., whose patients had a mean binocular UIVA of 0.32 (±0.15) logMAR (at 70 cm); the postoperative monocular UIVA was 0.45 (±0.18) logMAR in the group of patients with trifocal lens Finevision Micro F IOL (Physiol) [21]. According to Kohnen et al., the mean binocular UIVA after AT Lisa tri IOL implantation was 0.00 (±0.12) logMAR [22].

Although it is widely recognized that presurgery CS is undoubtedly affected by the lens opacity, we would like to emphasize that, in our study, the postsurgery logarithmic values of parameters that affect CS were significantly higher than those revealed by the presurgery testing. No significant differences were found in subsequent postoperative follow-ups indicating stability of CS function. Higher contrast sensitivity was observed at the frequencies of 3 cpd and 6 cpd compared to 1.5, 12, and 18 cpd. Numerous studies have provided normative data for contrast sensitivity functions in various age groups of the healthy population [26–28]. The results of our WIOL-CF group were comparable to those characteristic of the age group from 60 years and onwards [27]. Contrast sensitivity changes in patients with bioanalogic lenses presented in this study are the first in ophthalmic literature. Comparisons of contrast sensitivity outcomes pose problems due to a wide variety of testing methods, lighting levels, and inconsistencies in the reported variables. Alio et al. analyzed 132 studies, 31 of which presented comparisons between multifocal and monofocal IOL groups. One-third of these studies showed that there was no difference in the CS function between these groups. The remaining studies, however, indicated some CS decline at higher frequencies of multifocal lenses compared to the group of monofocal lenses [4]. CS for the diffractive multifocal lenses Restor (Alcon) showed lower values at all spatial frequencies compared to monofocal lenses [29]. Contrast sensitivity in patients with 1CU lenses and monofocal lenses did not differ significantly in randomized studies by Harman et al. and Kamppeter et al. [30,31]. Similar results were obtained by other authors [23,32]. Alio et al. also compared the quality of vision between three groups of binocularly implanted multifocal lenses, i.e., bifocal refractive-diffractive AT Lisa IOL (Carl Zeiss), trifocal AT Lisa IOL (Carl Zeiss), and apodized bifocal IOL ReSTOR (Alcon). There were no statistically significant differences between the three IOL groups for the contrast scores at one and six months after surgery. Groups with trifocal and bifocals lenses showed similar CS in all tested spatial frequencies (*p* ≥ 0.053) [33]. Similar results were described by Jonker et al., who compared trifocal Finevision Micro F IOL (PhysIOL SA) with bifocal Acrysof IQ Restor + 3.0D IOL (Alcon) [21]. Pedrotti et al. compared 4 IOL models (1 monofocal IOL, 2 multifocal IOLs, and 1 EDOF). They found no significant differences in contrast sensitivity under photopic conditions between monofocal IOL and EDOF IOL (*p* = 0.293). The CS values for these two IOL groups were significantly better than those obtained with the two groups of apodized diffractive-refractive IOLs (+2.5 D MIOL, +3.0 D MIOL) (*p* = 0.001) [24]. We also evaluated high-order aberration RMS (HOA-RMS) and total order aberration RMS (TOA-RMS) using the WASCA aberrometer. An analysis of changes in higher-order aberration showed significant differences between presurgery and postsurgery means; the results were significantly lower at the last follow-up. Comparable postoperative results were obtained by the Czech team of Siatiri et al. with the following means: TOA-RMS of 2.75 (±1.66) *μ*m and HOA-RMS of 1.08 (±0.48) *μ*m. [17]. The authors used i-Trace aberrometer to measure higher aberrations after implantation of WIOL-CF. It should be noted that the Tracey Technologies Inc. (Houston, Tex) system has comparable efficiency in measuring spherical aberrations to the WaveScan system (Hartmann-Shack) [34]. The above TOA- and HOA-RMS can be compared to optical aberrations in pseudophakic eyes with different IOL types [35,36]. In a study of aspherical, monofocal IOLs, Kretz et al. obtained the following posttreatment means: TOA-RMS of 1.74 (±0.98) *μ*m and HOA-RMS of 0.51 (±0.19) *μ*m [37]. The mean HOA-RMS obtained by Pallikaris et al. using the WASCA aberrometer was lower, i.e., −0.18 (±0.13) *μ*m, ranging from 0.02 to −0.5 *μ*m at one year after the procedure [14]. In our study group, the defocus curve of the bioanalogic lens at the three main VA levels did not exceed the line corresponding to 0.3 logMAR. The analysis of changes in the patient's subjective satisfaction assessed using the VF-14 test and the spectacles independence test showed a significant improvement in the study parameters. Approximately 72% of Pallikaris et al.' patients could see at near distances without spectacles [14]. There were no significant differences between EDOF (mean 77.94 ± 25.72) and +3.0 D MIOL (mean 69.25 ± 24.57) in the study by Pedrotti et al. [24]. Kohnen et al. reported that 88% of patients achieved spectacle independence after binocular AT Lisa tri 839 MP lens implantation [22]. We did not observe WIOL-CF lens dislocation, including eyes after YAG capsulotomy due to opacity of the posterior capsule. There are, however, some reports on bioanalogic lens dislocation in Korean patients [38–40]. The late postoperative complications that occurred in our study group included posterior bag opacification (PCO) in 6 eyes (12%). Two patients (3 eyes, 6%) required Nd: laser capsulotomy resulting in improved VA. Pallikaris et al. reported no intraoperative and early postoperative complications in the study group. The PCO value was lower than in this study, i.e., 4% (2/50 eyes) in the 12-month follow-up. One patient underwent YAG capsulotomy; no complications related to lens geometry were noted [14].

A limitation of this study is the lack of a control group, which is a common limitation in a retrospective case series study design. Another limitation is a small sample size. However, binocular interventions certainly help assess visual acuity outcomes with this type of IOLs.

## 5. Conclusions

The evaluation of a WIOL-CF bioanalogic intraocular lens showed good *distance*, *intermediate*, and *near* visual acuity. Contrast sensitivity increased after surgery in all spatial frequencies. Patient satisfaction was high despite some optical phenomena. The rate of postoperative spectacle independence also turned out high.

## Figures and Tables

**Table 1:** Postoperative spherical equivalent refractive outcome (D).

Postoperative SE refractive outcomes (D)	−0.25 to +0.25	−0.5 to +0.5	−1.0 to +1.0	−1.5 to +1.5	−2.0 to +2.0
Number of eyes	14	26	40	47	49
Percentage of eyes	28	52	80	94	98

**Table 2:** Visual outcomes during the preoperative examination and the follow-up visits.

Parameter (mean ± SD)	PRE pre-op	2 WEEKS	3 MTH	6 MTH	12–24 MTH	24–36 MTH	*p* value∗	*p* value^
UDVA Snellen	0.375 ± 0.259	0.962 ± 0.095	0.983 ± 0.115	1.00 ± 0.149	1.004 ± 0.131	1.008 ± 0.168	<0.001	0.406
BCDVA Snellen	0.629 ± 0.324	1.024 ± 0.089	1.038 ± 0.087	1.065 ± 0.149	1.061 ± 0.122	1.056 ± 0.171	<0.001	0.33
UNVA Jeager 40	5.158 ± 4.371	4.08 ± 2.465	3.542 ± 2.01	3.521 ± 1.81	3.714 ± 1.947	3.359 ± 1.77	0.537	0.051
UIVA Jeager 70	4.974 ± 3.752	3.100 ± 2.435	3.063 ± 2.187	3.021 ± 2.088	2.918 ± 1.88	2.538 ± 1.62	0.005	0.293

UDVA = uncorrected distance visual acuity; BCDVA = best-corrected distance visual acuity; UNVA = uncorrected near visual acuity; UIVA = uncorrected intermediate visual acuity; SD = standard deviation. ∗Comparison between pre-op and postoperative measurement. ^Comparison between periods after operation.

**Table 3:** Binocular visual outcomes during the preoperative examination and the follow-up visits.

Binocular (mean ± sd)	PRE pre-op	2 Weeks	3 MTH	6 MTH	12–24 MTH	24–36 MTH	*p* value∗	*p* value^
UDVA Snellen	0.482 ± 0.276	1.016 ± 0.114	1.054 ± 0.15	1.054 ± 0.122	1.083 ± 0.176	1.089 ± 0.251	<0.001	0.138
BCDVA Snellen	0.772 ± 0.239	1.032 ± 0.075	1.1 ± 0.153	1.096 ± 0.155	1.133 ± 0.188	1.132 ± 0.25	<0.001	0.118
UNVA Jeager 40	—	2.76 ± 1.855	2.583 ± 1.64	2.917 ± 1.586	3.083 ± 1.67	2.737 ± 1.447	—	0.6
BCNVA Jeager 40	—	1.24 ± 0.436	1.25 ± 0.442	1.333 ± 0.415	1.208 ± 0.415	1.474 ± 0.612	—	0.3
UIVA Jeager 70	—	2.28 ± 1.696	2.7 ± 1.967	2.25 ± 1.22	2.25 ± 1.22	2.053 ± 1.268	—	0.317
BIVA Jeager 70	—	1.640 ± 1.036	1.75 ± 1.073	1.625 ± 0.647	1.417 ± 0.584	1.316 ± 0.582	—	0.011

UDVA = uncorrected distance visual acuity; BCDVA = best-corrected distance visual acuity; UNVA = uncorrected near visual acuity; BCNVA = best-corrected near visual acuity; UIVA = uncorrected intermediate visual acuity; BIVA = best-corrected intermediate visual acuity; SD = standard deviation. ∗Comparison between pre-op and postoperative measurement. ^Comparison between periods after operation.

**Table 4:** The mean contrast sensitivity outcomes before and after the surgery in the WIOL-CF group.

Group	CS [LogCS]							
	Qualification				24–36 months after			
	Mean	SD	Min	Max	Mean	SD	Min	Max
A [1.5 cpd]	**0.873**	0.579	0.045	1.56	**1.42**	0.199	1.11	1.85
B [3.0 cpd]	**1.079**	0.362	0.7	1.76	**1.731**	0.194	1.46	2.06
C [6.0 cpd]	**1.117**	0.346	0.78	1.81	**1.669**	0.21	1.2	2.11
D [12.0 cpd]	**0.82**	0.281	0.6	1.340	**1.134**	0.298	0.6	1.63
E [18.0 cpd]	**0.411**	0.218	0.17	1.08	**0.591**	0.336	0.3	1.52

**Table 5:** High-order aberration and total aberration (WASCA) before and after surgery.

Parameter	PRE	2WEEKS	3 MTH	6 MTH	12–24 MTH	24–36 MTH	*p* value^∗^	*p* value^
HOA-RMS (*μ*m)	6.011 ± 17.811	1.335 ± 5.854	0.571 ± 0.228	0.565 ± 0.202	1.754 ± 7.939	0.684 ± 0.228	0.018	0.002
TOA-RMS (*μ*m)	7.973 ± 23.821	2.249 ± 6.215	1.629 ± 0.719	1.819 ± 0.757	1.818 ± 0.894	2.015 ± 0.796	0.00019	0.0015

∗Comparison between pre-op and postoperative measurement. ^Comparison between periods after operation.

## Data Availability

The data used to support the findings of this study are available from the corresponding author upon reasonable request.
